# Biventricular unloading and rhythm control leading to biventricular recovery: a case report

**DOI:** 10.1093/ehjcr/ytaf536

**Published:** 2025-10-21

**Authors:** María de Miguel-Alava, Corry O’Sullivan, Christophe Vandenbriele, Mohamed Osman, Fernando Riesgo-Gil

**Affiliations:** Department of Cardiology, University Hospital of Valladolid, Ramón y Cajal 3, Valladolid 47003, Spain; Institute of Heart Sciences (ICICOR), Ramon y Cajal 3, Valladolid 47003, Spain; Health Research Institute of Valladolid (IBioVALL), Ramon y Cajal 3, Valladolid 47003, Spain; CIBER CV. Health Institute Carlos III. Monforte de Lemos 3-5, 28029 Madrid, Spain; Department of Heart Failure, Transplantation and Mechanical Circulatory Support, Royal Brompton and Harefield NHS Foundation Trust, Hill End Rd, Harefield, Uxbridge UB9 6JH, London, UK; Department of Cancer and Surgery, Imperial College, Exhibition Rd, South Kensington, London SW7 2AZ, UK; Heart Center Aalst, Moorselbaan 164, Aalst 9300, Belgium; Department of Adult Intensive Care, Royal Brompton and Harefield NHS Foundation Trust, Hill End Rd, Harefield, Uxbridge UB9 6JH, London, UK; Department of Cardiothoracic and Transplant Surgery, Royal Brompton and Harefield NHS Foundation Trust, Hill End Rd, Harefield, Uxbridge UB9 6JH, London, UK; Clinical Lead for Cardiology Transplant Medicine, Department of Heart Failure, Transplantation and Mechanical Circulatory Support, Royal Brompton and Harefield NHS Foundation Trust, Hill End Rd, Harefield, Uxbridge UB9 6JH, London, UK

**Keywords:** Biventricular unloading, Cardiogenic shock, Case report, Dilated cardiomyopathy, Heart failure, Left ventricular reverse remodelling, Mechanical circulatory support

## Abstract

**Background:**

Temporary mechanical circulatory support (tMCS) is a life-saving intervention in cardiogenic shock (CS), though not without risks. Increasing attention has been given to ventricular unloading and its role in myocardial recovery, particularly in reversible cardiomyopathies.

**Case summary:**

A 35-year-old man presenting with CS was initially treated with veno-arterial extracorporeal membrane oxygenation (V-A ECMO) and Impella (ECPELLA). Due to complications, support was escalated to Levitronix biventricular assist device. After 5 months of support and optimized medical therapy, substantial myocardial recovery enabled weaning from MCS and successful hospital discharge.

**Discussion:**

MCS provides critical time to investigate the underlying cause of CS and assess the potential for recovery. This case illustrates how MCS can be both life-saving and a platform for ventricular unloading and neurohormonal optimization. Although full recovery is rare in severe cases, such strategies may improve cardiac function and enable successful weaning from support.

Learning points

- Early biventricular unloading combined with guideline-directed medical therapy can facilitate meaningful myocardial recovery, even in severe cardiogenic shock.
- Identifying candidates for recovery remains complex and requires integrating clinical, haemodynamic, and patient-specific factors during MCS support and weaning.
- A genetic predisposition should be considered even when HF appears to be triggered by an identifiable event.

## Introduction

Mechanical circulatory support (MCS) has enabled clinicians to rescue patients with cardiogenic shock (CS) and cardiac arrest while also allowing time to assess myocardial recovery and underlying aetiologies.

Moreover, when applied with sound clinical judgment, ventricular unloading and the opportunity to initiate or optimize neurohormonal therapy can provide substantial benefits. MCS serves as a bridge to transplantation, transition to durable left ventricular assist device (LVAD), or—as in the rare case we present—facilitate full ventricular recovery, resolution of MCS-related complications, and successful hospital discharge.

Our case is unique in that biventricular unloading was achieved using the Levitronix system, and while the time to cardiac functional recovery was prolonged, the outcome was ultimately favourable.

## Summary figure

**Figure:**
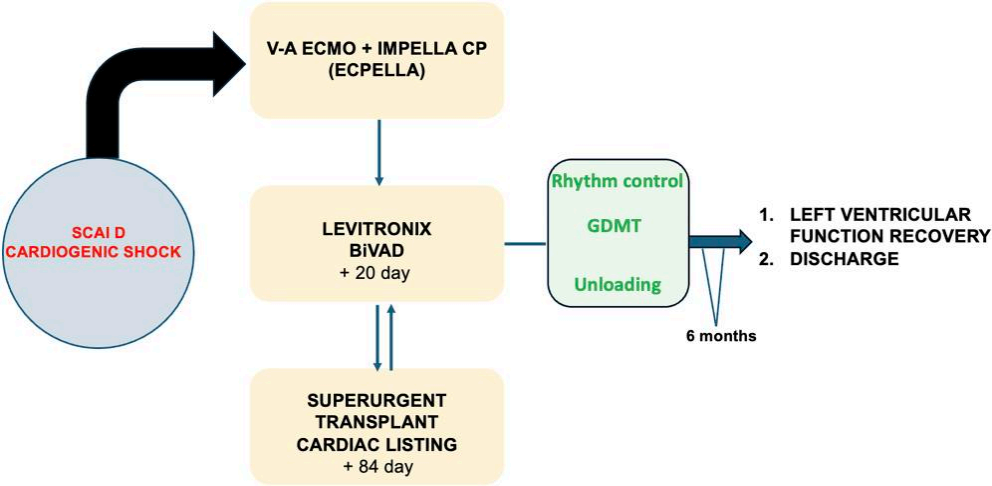


## Case presentation

A 35-year-old man with a prior heart failure (HF) episode related to atrial fibrillation (AF) in 2023, for which his general practitioner prescribed bisoprolol for rate control, presented in March 2024 with 5 days of worsening dyspnoea [New York Heart Association (NYHA) Class IV] and palpitations, following months of reduced exercise tolerance.

On admission, he was alert but slightly drowsy (Glasgow Coma Scale 15), with bilateral crackles, peripheral oedema (3+), hypotension (85/50 mmHg), and high central venous pressure (CVP, 24 mmHg). Oxygen saturation on room air was 90%.

**Figure 1:**
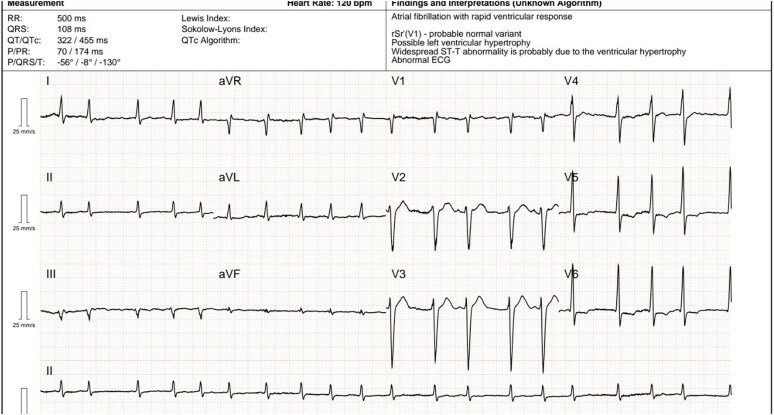
Electrocardiogram (25 mm/s, 10 mm/mV) on admission showing atrial fibrillation with a rapid ventricular response. QRS complexes are narrow with inverted T waves in leads V4–V6, a normal QTc interval, and a QRS axis of 0°.

Electrocardiogram (ECG) showed AF with a heart rate of 150 b.p.m. (*Figure 1*). Transthoracic echocardiography (TTE) showed severe biventricular dysfunction [left ventricular ejection fraction (LVEF) 20%] with severe right ventricular (RV) dilation and impairment (TAPSE 8 mm), biatrial enlargement, moderate mitral and tricuspid regurgitation, and a left ventricular (LV) outflow tract velocity time integral (VTI) of 5 cm. Laboratory results showed arterial lactate of 12 mmol/L, NTproBNP 23 000 pg/ml, troponin T 320 pg/ml, and evidence of multiorgan failure. CS was diagnosed.

He received digoxin, amiodarone, furosemide, and milrinone. With worsening status, a multidisciplinary team (MDT) initiated veno-arterial ECMO (V-A ECMO) as a bridge-to-decision. Concurrent renal replacement therapy (RRT) addressed fluid overload and metabolic derangements.

By Day 2, inadequate LV unloading and pulmonary oedema required non-invasive ventilation. An Impella CP micro-axial flow pump was placed for LV venting. Sinus rhythm (SR) was restored after 48 h of amiodarone without electrical cardioversion. Impella malposition led to haemolysis and thrombocytopenia; after failed repositioning, it was replaced.

On Day 9, heparin-induced thrombocytopenia (HIT) was diagnosed. Heparin was replaced with argatroban. Plasma exchange and intravenous immunoglobulin followed.

Due to limitations of ECMO—impairs accurate assessment of pulmonary artery pressures and pulmonary vascular resistance, hemocompatibility issues, suboptimal unloading for promoting recovery, and anticipated long transplant wait (blood group O, BMI >30)—a more durable and physiologically appropriate form of MCS was needed. Following HIT antibody negativity and given the severity of RV dysfunction, a Levitronix biventricular assist device (BiVAD) was chosen.

Implantation occurred on Day 20. The postoperative period was complicated by airway bleeding necessitating bronchoscopy, cryotherapy, and temporary oxygenator in the RV assist device circuit. Anticoagulation was withheld; transfusions and thromboelastography-guided factor management were required.

Despite these complications, he remained human leukocyte antigen panel-reactive antibody negative and experienced no thrombotic events. A 48-gene panel for dilated cardiomyopathy (CMP) was performed, yielding negative results. Endomyocardial biopsy samples showed no evidence of myocarditis or infiltrative disease. The genetic testing was carried out at the Clinical Genetics and Genomics Laboratory of a British Hospital using Illumina NGS platforms (MiSeq/NextSeq 550), covering all coding regions with a minimum coverage of 20×. Variants of uncertain significance were reported; however, in the absence of relevant family history, these variants were considered incidental findings without clinical significance.

Over the following weeks:

1. Haemostasis allowed anticoagulation reintroduction (argatroban).
2. RRT was reduced and stopped.
3. He was weaned from ventilation and decannulated post-tracheostomy.
4. Guideline-directed medical therapy (GDMT) was initiated with an angiotensin-converting enzyme inhibitor given the patient’s tendency towards elevated blood pressure. A mineralocorticoid receptor antagonist and an SGLT2 inhibitor were subsequently added, as the estimated glomerular filtration rate (eGFR) was above 30 ml/min/1.73 m², and the drugs are well tolerated. Finally, a beta-blocker was introduced with caution, as its negative inotropic effect was considered the most challenging step in the titration process. SR was maintained with amiodarone.
5. Functional improvement led to surgical transplant listing candidacy.

On Day 84, he was listed for super-urgent listing due to persistent BiVAD dependence and poor function. Surprisingly, after 67 days, TTE showed LVEF improvement. A right heart catheterization with staged BiVAD flow reduction confirmed stable hemodynamics, suggesting suitability for weaning.

BiVAD support was gradually reduced under TTE guidance. On Day 154, he was taken to theatre. With flows reduced to 1 L/min and clamping of cannulae, tolerance was confirmed (CVP 8 mmHg, wedge pressure 15 mmHg, Fick cardiac index >2.2 L/min/m²), and the system was explanted via cardiopulmonary bypass using existing right atrial and aortic BiVAD cannulae.

**Figure 2:**
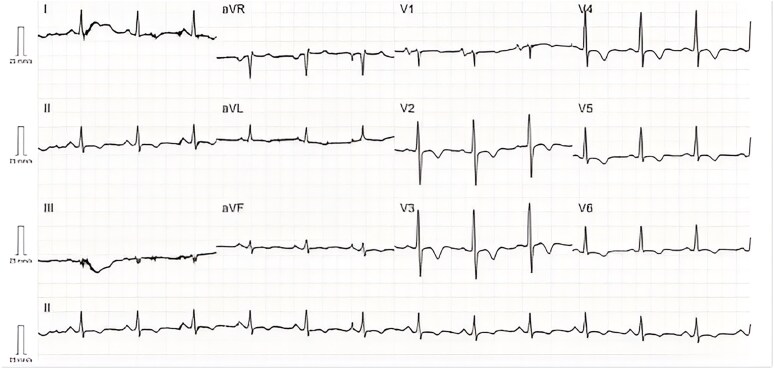
Electrocardiogram (25 mm/s, 10 mm/mV) at discharge. Sinus rhythm at 80 b.p.m., normal PR interval, narrow QRS complexes, inverted T waves in V2–V6, I, II, aVF, and aVL. Normal QTc Interval. QRS axis at 0°.

He was discharged home with SR (*Figure 2*), NYHA Class I, and on complete GDMT (eplerenone 50 mg/24 h, enalapril 20 mg/12 h, dapagliflozin 10 mg/24 h, and bisoprolol 5 mg/12 h). He expressed deep gratitude, stating he had lost hope but was now optimistic.

Pre-discharge TTE showed a LVEF 40%, mild RV dysfunction (TAPSE 13 mm), and no significant valvular disease. Outpatient cardiac magnetic resonance imaging and AF ablation were scheduled, but he relocated abroad. Telephone follow-up confirmed NYHA I status and normal daily activity.

## Discussion

When CMP is suspected, current guidelines recommend a multidisciplinary approach to identify reversible causes or underlying genetic conditions.^1^ However, when patients present with haemodynamic collapse and no prior personal or family history, as in our case, diagnosis and decision-making become particularly challenging. In such scenarios, MCS plays a critical role—not only to stabilize end-organ perfusion but also to allow time for diagnostic workup and therapeutic interventions.

Understanding the strengths and limitations of each MCS modality is crucial. Veno-arterial extracorporeal membrane oxygenation (V-A ECMO) restores systemic perfusion rapidly but increases afterload and complicates ventricular assessment. Intra-aortic balloon pumps offer minimal flow (<1 L/min) and are insufficient in severe biventricular failure. Impella devices (CP and 5.5) provide effective LV unloading but are limited by duration of use and are unsuitable for isolated right ventricular (RV) support. In contrast, biventricular systems such as the Levitronix offer prolonged support, complete biventricular unloading, and greater haemodynamic stability, making them ideal for patients awaiting transplant or demonstrating uncertain recovery potential.

MCS, as in our case, enables stabilization while reversible factors are addressed (e.g. tachyarrhythmias, electrolyte imbalances, ischaemia, hypothermia, or alcohol toxicity).^2,3^ In our patient, restoration of SR and metabolic correction did not lead to functional recovery, necessitating prolonged MCS.

Beyond simply allowing time for treatment, biventricular unloading may promote reverse cardiac remodelling. Although evidence is limited and largely based on observational data from univentricular devices like Impella, unloading shifts pressure-volume loops leftward and facilitates optimization of GDMT, often not feasible in patients with advanced HF and low cardiac index due to fragile haemodynamic balance, worsening end-organ function, and poor drug tolerance.^4–6^

Though tachycardia-induced CMP was likely the primary aetiology in our patient, delayed recovery suggests underlying myocardial vulnerability, and AF may have been a ‘second hit’. While genetic testing using a 48-gene panel did not identify known pathogenic mutations, a genetic predisposition cannot be ruled out and may have contributed to the delayed recovery.^7^

This case illustrates the full potential of MCS not only to bridge to transplant but to recovery—even when improvement is unexpectedly delayed.

## Lead author biography


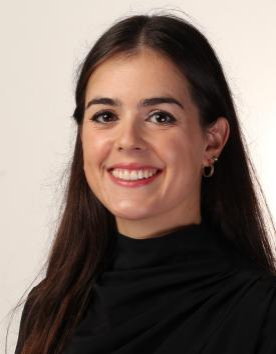


Dr María de Miguel-Alava graduated as an MD at Zaragoza University (Spain) in 2019. She is currently pursuing her specialization in Cardiology at the University Clinical Hospital of Valladolid. Her primary areas of interest include acute cardiovascular care and advanced heart failure. Dr. de Miguel-Alava completed a 4-month observership at Harefield Hospital, focusing on advanced cardiac care. She is expected to complete her cardiology training in September 2025.

## Supplementary Material

ytaf536_Supplementary_Data

## Data Availability

All relevant data are included in the article. No additional data are available.

## Supplementary material

Supplementary material is available at *European Heart Journal – Case Reports* online. Transthoracic echocardiographic findings at admission and at discharge are presented in Supplementary material online, *Videos S1* and *S2*, respectively.
